# The cross-scale correlations between individuals and nations in COVID-19 mortality

**DOI:** 10.1038/s41598-022-18179-8

**Published:** 2022-08-16

**Authors:** Lei Zhang, Yu-Rong She, Guang-Hui She, Rong Li, Zhen-Su She

**Affiliations:** 1 Institute of Health System Engineering, College of Engineering, Peking University, Beijing, China; 2 State Key Laboratory for Turbulence and Complex Systems, Peking University, Beijing, China

**Keywords:** Applied mathematics, Public health

## Abstract

It is challenging to quantitatively clarify the determining medical and social factors of COVID-19 mortality, which varied by 2 to 3 orders of magnitude across countries. Here, we present evidence that the temporal evolution of mortality follows a logistic law for 54 countries in four waves. A universal linear law is found between the early mortality growth time and the epidemic duration, one of the most important quantities, with a factor of 7.3 confirmed by data. Saturation mortality is found to have a power law relationship with median age and bed occupancy, which quantitatively explains the great variation in mortality based on the two key thresholds of median age (= 38) and bed occupancy (= 22%). We predict that deaths will be reduced by 38.5% when the number of beds is doubled for countries with older populations. Facing the next wave of the epidemic, this model can make early predictions on the epidemic duration and hospital bed demand.

## Introduction

Since COVID-19 was declared a pandemic by the World Health Organization (WHO) on March 11, 2020, approximately 5 million deaths have been reported across 184 countries or regions as of October 1, 2021. Therefore, lessons learned from the past waves, such as quantitative assessment of the severity of the epidemic and clarification of the determining medical and social factors, are urgently needed to help policymakers prevent more deaths in the next wave. Reliable death statistics and quantitative modelling are essential for understanding the pandemic^1^. However, it is a great challenge to quantitatively interpret observational data presenting a high variation in mortality evolution across countries, which reaches two to three orders of magnitude for the difference in case fatality rate (deaths per confirmed case) and crude death rate (deaths per 100,000 population). We attempt to propose a reliable understanding of this observation.

Previous studies have not yet reached a conclusion regarding this issue because they focused only on the individual scale or the national scale and ignored the cross-scale correlation. Specifically, patient-level studies have shown that older patients, men, and patients with underlying diseases have a greater risk of death and require respiratory assistance in the intensive care unit (ICU)^2–7^. However, the demographics of confirmed cases are changing over time, with more young people being infected in the later stages than in the early stages of the outbreak. On the other hand, country-level studies have suggested that healthcare resource availability, infection scale, etc., are associated with mortality^8–11^. However, countries’ capacity to prevent, detect, and respond to the outbreak varies widely over time^12^, so COVID-19 mortality presents great heterogeneity in space (across countries) and time^13^. Therefore, the commonly used method of a cross-sectional study, in which the mortality data for a given day are selected, yields a misleading representation by comparing the mortality data at different evolutionary stages. For example, studies concluded differently on whether there was a correlation between the case fatality rate and the number of tests^8,9,14–16^. Moreover, these various factors analysed at individual or national scale must form a coherent view to offer effective guidance for fighting against the pandemic.

**Figure 1:**
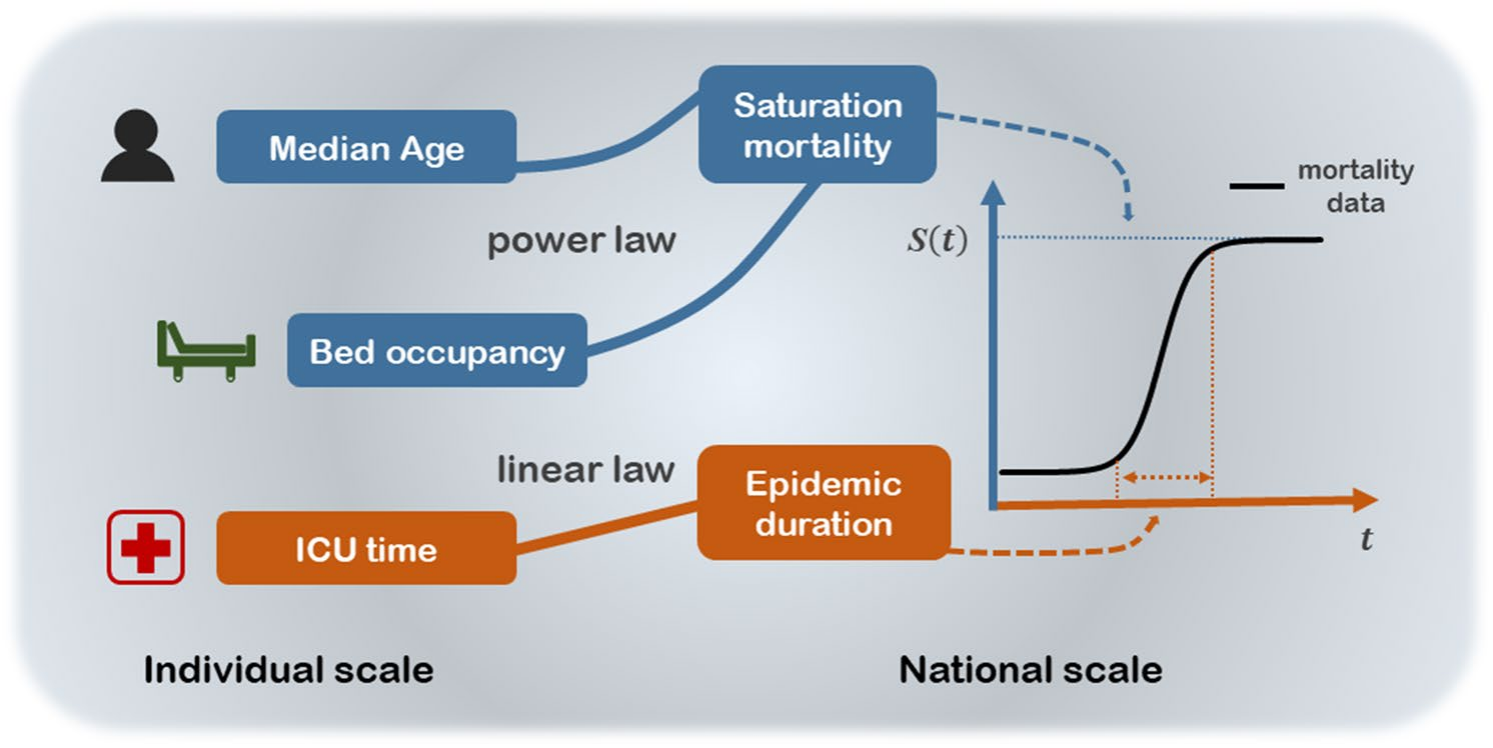
The cross-scale correlation between the individual scale and the national scale in this paper.

In sharp contrast to the above-mentioned studies, we adopt a dynamic study with a complete description of the complete evolution of mortality to better uncover robust features and explain the underlying mechanisms, which yields a quantitative prediction about future epidemic evolution. Considering that almost all countries have already experienced the 2 or 3 waves of the epidemic, it is time to derive a law governing the dynamics of COVID-19 mortality based on the data. Here, we present a logistic model that accurately describes the complete evolutionary patterns of COVID-19 mortality (deaths per 100,000 population, $S(t)$) across 54 countries, quantitatively explaining the great variation in mortality based on two key thresholds from exact scales of the median age (= 38) and bed occupancy (= 22%), finding the cross-scale correlations between the early mortality growth time ($\tau$) and epidemic duration with a dimensionless coefficient $k$. This time is also found, for several states with available data, to be very close to reported non-survival ICU time, which is a time of rebuttal against the virus at the individual level. As a result, cross-scale correlation analysis between individual and national scales is achieved in the first wave of COVID-19 mortality (see Fig. 1).

**Figure 2:**
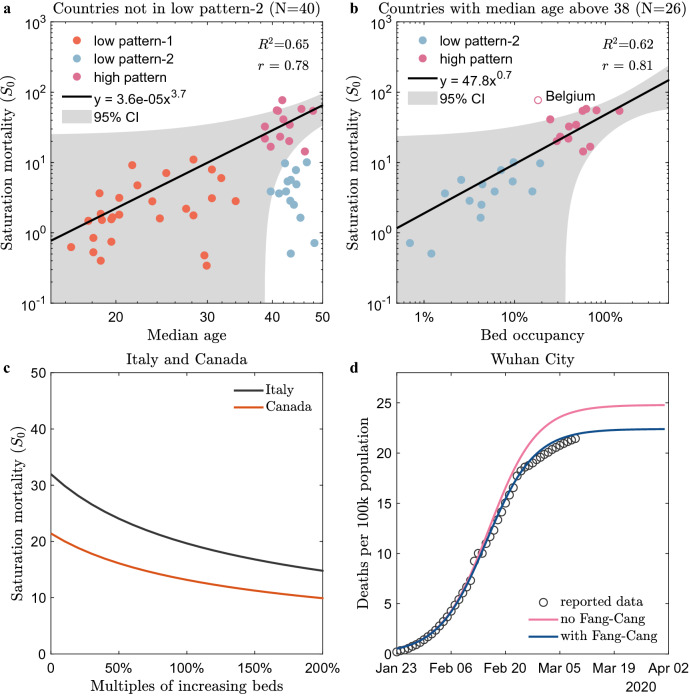
The mortality variation is explained by the median age and bed occupancy in the first wave. (**a**) The scaling between saturation mortality and the median age for countries without low pattern-2 ($N=40$). (**b**) The scaling between saturation mortality and peak bed occupancy for countries aged over 38 years old except Belgium ($N=26$). (**a**, **b**) take the double logarithmic coordinates for a better display of the data. The orange, blue and pink dots correspond to the low pattern-1, low pattern-2, and high pattern, respectively. The black lines are the fitting predictions by power functions. The grey areas are the 95% confidence intervals. Note that the red circle furthest from the confidence interval in (**b**) represents Belgium, probably because it uses a broader inclusion criterion for COVID-19 deaths^18^. (**c**) Reduction in saturation mortality when changing the number of beds. The black and orange lines represent Italy and Canada, respectively. (**d**) Contribution of the construction of Fang Cang hospital to the reduction in mortality in Wuhan City (Hubei Province, China). The grey circles are the official reported data. The red line represents the simulation of mortality evolution without Fang Cang hospital, while the blue line represents the simulation of mortality evolution with Fang Cang hospital. ${R}^{2}$ is the goodness of fit and $r$ is the Pearson correlation coefficient.

**Figure 3:**
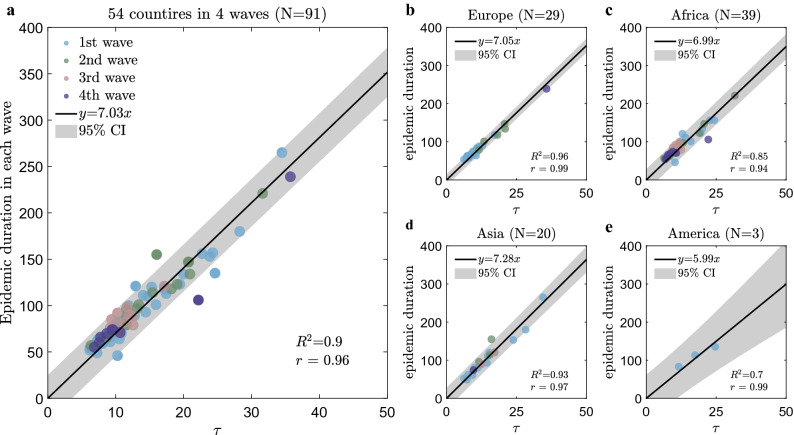
The cross-scale correlation between $\tau$ and epidemic duration in four waves. (**a**–**e**) are the correlations with different but similar correlation coefficients $k$ for all countries, Europe, Africa, Asia, and America, respectively. The abscissa variable is the fitting parameter $\tau$, which is the characteristic time for early mortality growth. The ordinate variable is the duration of four waves. The dots are the data across countries in each wave. The black lines are the fitting predictions by linear functions. The grey areas are the 95% confidence intervals. ${R}^{2}$ is the goodness of fit and $r$ is the Pearson correlation coefficient.

**Figure 4:**
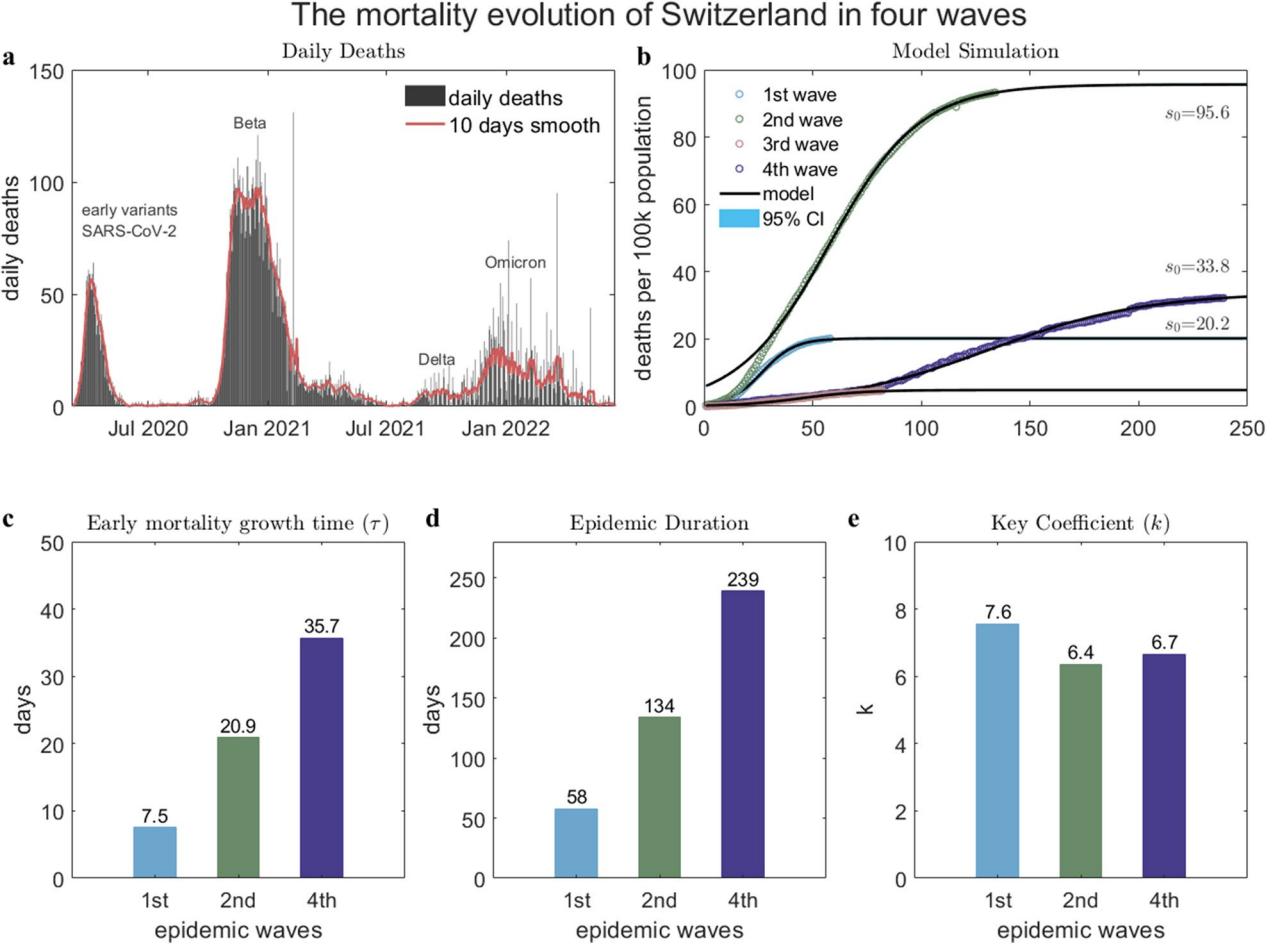
The mortality evolution of Switzerland in four waves. (**a**) The reported daily deaths data. The red line is the 10-day smoothed results. (**b**) The model simulation of the mortality in each wave. (**c**) The early mortality growth time ($\tau$) in each wave. (**d**) The epidemic duration in each wave. (**e**) The key coefficient $k$ in each wave. Note that the third wave is not discussed in (**c**), (**d**), and (**e**), because it did not have a complete evolution.

Specifically, the data show that, for all countries with substantial death (above 100), the temporal evolution of mortality during the first pandemic wave well fits a logistic pattern with just two parameters (see “Methods” for details), which allows decoupling the complex evolutionary behaviour into two independent processes with close medical and social correspondences. One is saturation mortality (${s}_{0}$) in the late stage of the epidemic, which is positively correlated with the state’s median age and bed occupancy (see Fig. 2a,b). This finding allows us to derive, from data, a law that yields a prediction of practical interest, namely, how many hospital beds need to be reserved in the continuing fight against the next wave of the epidemic if the aim is to cut the number of deaths by half (see Fig. 2c,d). The other is a characteristic growth time ($\tau$), which is closely related to the ICU time in the early stage and shows a universal linear correlation with epidemic duration across different countries in four waves (see Figs. 3, 4). If this is further confirmed, it would be possible to predict the epidemic duration from collected clinical data (i.e. non-survival ICU time) at the early stage of the outbreak (see Table 1). Thus, we highly recommend testing the current model, which, if successful, would greatly enhance our ability to understand and predict the current epidemic.

**Table 1:** The predictions of epidemic duration by using $k=7.3$ in the first wave.

Country/region	$\tau$ (days)	Predicted duration (days)	Actual duration (days)	ICU time* (days)
Wuhan	6.2	45	51	7
Germany	9.5	69	73	9
Italy	11.6	85	87	10
Denmark	9.8	72	70	7.02
Greece	10.9	80	84	8

*Data are expressed as the median in Refs.^19–23^.

## Results

### Two key thresholds associated with age, beds, and saturation mortality (${{\varvec{s}}}_{0}$)

When the epidemic ends, the mortality $S(t)$ evolves to ${s}_{0}$, so the saturation mortality parameter ${s}_{0}$ quantifies the epidemic. Previous published cross-sectional studies^3–7^ did show a correlation between age, hospital beds, and mortality, but it is not clear which role these factors play in different countries and at different stages of the outbreak. In Fig. 2, we plot ${s}_{0}$ as a function of the nation’s median age and available hospital beds, which immediately reveals some remarkable features. First, the high-mortality patterns, which are defined that the ${s}_{0}$ are larger than the average value $\bar{{s }_{0}}$ (12.0), were all found in countries with a median age over 38 years old, suggesting that median age is one of the key factors influencing mortality, consistent with previous studies^3–7^. The current finding is more quantitative: it presents a scaling between the saturation mortality and median age, with a power law exponent of 3.7 (see Fig. 2a).

On the other hand, the deviation from this scaling is quite substantial for countries of high median age (most blue dots are outside of the confidence interval, see Fig. 2a). The strong scattering suggests that there must be other important factors causing high mortality. Indeed, we found that there exists a nice scaling between saturation mortality and bed occupancy at the peak of the first wave (confirmed cases at peak time over bed number, see “Methods” for details) for countries with a median population age above 38 years old (see Fig. 2b). The saturation mortality increases with the peak bed occupancy, following a power law of exponent 0.7, which attributes the large variation in the mortality of older countries to the great variation in peak bed occupancy (almost by two orders of magnitude) across countries. The peak bed occupancy reflects the degree of countries’ medical support during the pandemic. It would be interesting to discover a critical value of the peak bed occupancy to separate low and high mortality. As shown in Fig. 2b, a value between 20 and 24%, e.g., 22%, may be a reference.

This power law predicts that the deaths will be reduced by approximately 38.5% when the number of beds is doubled (see Fig. 2c). In other words, increasing the number of beds is very effective in preventing deaths in older countries. Specifically, if Italy could double the number of beds, approximately 13,400 deaths could be prevented in the first wave, and similarly, approximately 3500 deaths could be prevented in Canada, as shown in Fig. 2c.

The above discussion on the cross-scale correlations in the first wave of COVID-19 mortality also yields a method for the quantitative evaluation of the efficacy of interventions and the prediction of epidemic duration. For example, Wuhan City (Hubei Province, China) is comparable in population size and pandemic scale to some countries. Its bed occupancy (at the peak) based on the number of beds before the outbreak was 39.2%, and the saturation mortality (${s}_{0}$) was predicted to be 24.8. However, after the construction of Fang Cang Hospital, the number of beds increased by 15,000^17^, and ${s}_{0}$ reduced to 22.4, which is very close to the actual saturation mortality of 21.9. Thus, the power law could explain a reduction of 82% (or 268 deaths) in the number of deaths, as shown in Fig. 2d.

In summary, the high variation in mortality across countries can be explained quantitatively. First, two key quantities of median age and bed occupancy can classify all countries into three typical patterns. Older countries (> 38) with high bed occupancy (> 22%) defined as the high pattern have far greater mortality than the other two low-mortality groups, which include younger groups (< 38) defined as the low pattern-1, without exception, and the group with low bed occupancy (abundant medical supplies) defined as the low pattern-2. What is interesting is that some older countries (> 38) can evolve into the low-mortality pattern if they have a low bed occupancy (< 22%). This indicates that bed occupancy is an essential indicator to evaluate the effectiveness of national anti-COVID-19 measures.

### The dimensionless coefficient ${\varvec{k}}$ between ${\varvec{\tau}}$ and epidemic duration

The present two-parameter model not only explained the large variations in mortality across countries but also discovered a cross-scale correlation between the early mortality growth time ($\tau$) and epidemic duration, which provides new insights regarding the spread and evolution of COVID-19. First, a dimensionless coefficient $k$ can be derived from our model (see “Methods” for details), which is the ratio of the epidemic duration to the model parameter $\tau$ (which coincides with the ICU time of non-survivors in the early stage of the epidemic). Importantly, we find that the $k$ values are nearly constant across different continents in all four waves, and the average value $\bar{k }$ is 7.3 (IQR 6.8–7.8), as shown in Fig. 3. This linear law is better obeyed for countries with shorter $\tau$ (< 20), while the data are more diffused for countries with longer $\tau$ (> 20) (see Fig. 3a). This suggests that $\tau$ is a good indicator of the “strength” of the epidemic, or how long it may last. The longer the $\tau$ is, the longer the epidemic will last.

Knowing the value of $k$, one may predict the duration of the outbreak by the estimate of $\tau$ in the early stage. Using the average value $\bar{k }=7.3$ and the early mortality growth time ($\tau$), the durations are predicted and shown to be very close to the actual values, as presented in Table 1. As a result, policymakers can more accurately anticipate the difficulties of the fight against the pandemic and better optimize the load on the health system to minimize the number of deaths.

Note that $\tau$ is also found, for several states with available data, to be very close to reported non-survival ICU time in the early stage, as compared to published clinical data^19–23^ (see Table 1). This indicates the importance of the ICU time of non-survivors in the early stage, which is often overlooked in clinical studies; the latter is related to an individual’s antiviral ability, while the duration of the epidemic is a social scale anti-epidemic effect. Currently, the ICU time is a clinical statistic with predictive accuracy greatly limited by the samples, we call for more accurate statistical studies of this critical indicator, which is sparsely reported now. Thus, this linear law reveals an important property of COVID-19, which needs further investigation.

Take the case of Switzerland, for example, which has complete 1st, 2nd, and 4th waves data showing considerable variation (see Fig. 4a). Our model provides a good description of all data, and in addition, reveals some underlying evolutionary mechanisms. First, the saturation mortality ${s}_{0}$ quantifies the severity of the second versus first wave, by a factor of 4.7 (see Fig. 4b), while the fourth wave is more severe than the first wave by a factor of 1.7. Secondly, the early mortality growth time $\tau$ is found to increase, showing a decline in the toxicity of the virus, which is consistent with clinical studies^24^. The currently prevalent Omicron strain has a $\tau$ which is 4.8 times longer than the first wave, and 1.7 times longer than the second wave (see Fig. 4c), while its epidemic duration is 4.1 times longer than the first wave and 1.8 times longer than the second wave (see Fig. 4d). One then finds an approximately constant ratio $k$ between the early mortality growth time ($\tau$) and epidemic duration (see Fig. 4e), which has been verified above through 54 countries in four epidemic waves.

## Discussion

The COVID-19 pandemic has forced us to rethink the way countries prepare for public health crises. Although the end of the crisis is not yet in sight, it is time to learn from data the rules governing the evolution of the epidemic, which may help to improve the global capacity to respond to health crises of this magnitude. This becomes possible since, for the first time in the history of science, so much information has been collected and shared worldwide, so that one may gain a deeper understanding of epidemic evolution^25^.

The present study demonstrates the validity of a logistic model to describe the complete evolution of COVID-19 mortality in the first wave across 54 countries. We then find, from the data, a remarkable correlation for epidemic characteristics between the individual scale (age and non-survival ICU time) and national scale (global mortality and epidemic duration). This correlation quantitatively answers two crucial questions of major interest, namely, why does COVID-19 mortality vary so widely from country to country, and how long will the epidemic last?

For the first question, the power-law correlation between the median age, bed occupancy, and saturation mortality yields a quantitative explanation for the large observed variation in mortality across countries (see Fig. 2), with two key thresholds, 38 years old for median age and 22% for bed occupancy, which classifies countries into three typical patterns. Specifically, older countries could flatten the mortality curve by reducing bed occupancy to under 22%, offering an impressive model for other countries in reducing deaths in the next wave.

For the second question, the linear law between the two different time scales makes it possible to predict the duration of the epidemic wave by using the early mortality growth time ($\tau$) and the key coefficient $k$ (see Fig. 3, Table 1). It is well known that the long-term forecasting of outbreaks is a great challenge since there are so many unpredictable and complex factors, such as virus mutations, policy changes, etc., that can influence epidemic evolution. But, the discovered linear relation between $\tau$ and the epidemic duration (as a consequence of the logistic law) suggests the existence of a global organization law governing the human fight against the epidemic. Natural, some countries display deviations from the linear law with much longer epidemic duration, and the reason behind this deserves more careful study in the future (see Fig. 3). For instance, it is interesting to verify and compare the values of the dimensionless coefficient $k$ with the values in other epidemics, such as SARS or the annual flu, which may bring new insights into understanding the spread and evolution of the epidemic.

An important finding of the present study is the possible relation between $\tau$ and the ICU time of non-survivors in the early stage, which is often overlooked in clinical studies. We found that Wuhan City had the smallest $\tau$ (6.2 days) among all the regions included in the study, which was very close to the 7 days of the clinical study^19^ (see Table 1). This result is consistent with the fact that Wuhan City was the first outbreak point so the virus is more toxic, and that the community knew little about the virus at the beginning of COVID-19. Moreover, the wide range of $\tau$ across countries (see Fig. 3) needs to be studied and explained in the future, and the current findings suggest collecting more accurate ICU time data for better prediction of the next wave of the pandemic.

Our study still has several limitations. First, the data analysed here are from public databases. The limited quality, different statistical standards, and incompleteness of databases may affect the precision of our description. Second, our study does not include countries with complex evolutionary behaviours, such as repeated rebounding of the outbreak. Third, we used median age data at the national level instead of at the patient level; the latter is currently lacking in most countries. Finally, we use the number of beds before the outbreak instead of the actual data that tend to increase during the epidemic, which is currently difficult to obtain. Although these factors influence the precision of the description, they would not affect the main conclusions of the study for the first wave of COVID-19 in most countries.

In conclusion, we present a dynamic model that decouples the complex evolution of COVID-19 mortality into two key processes: the mortality growth time in the early stage and the saturation mortality in the late stage. This analysis uncovers three cross-correlations between the individual scale and the national scale in COVID-19 mortality, which enables us to evaluate interventions quantitatively and predict the epidemic duration. This framework also has other potential applications, such as providing country-specific suggestions for the reservation of hospital beds to fight against the next wave of COVID-19 so that more deaths can be prevented.

## Methods

### Study design

**Figure 5:**
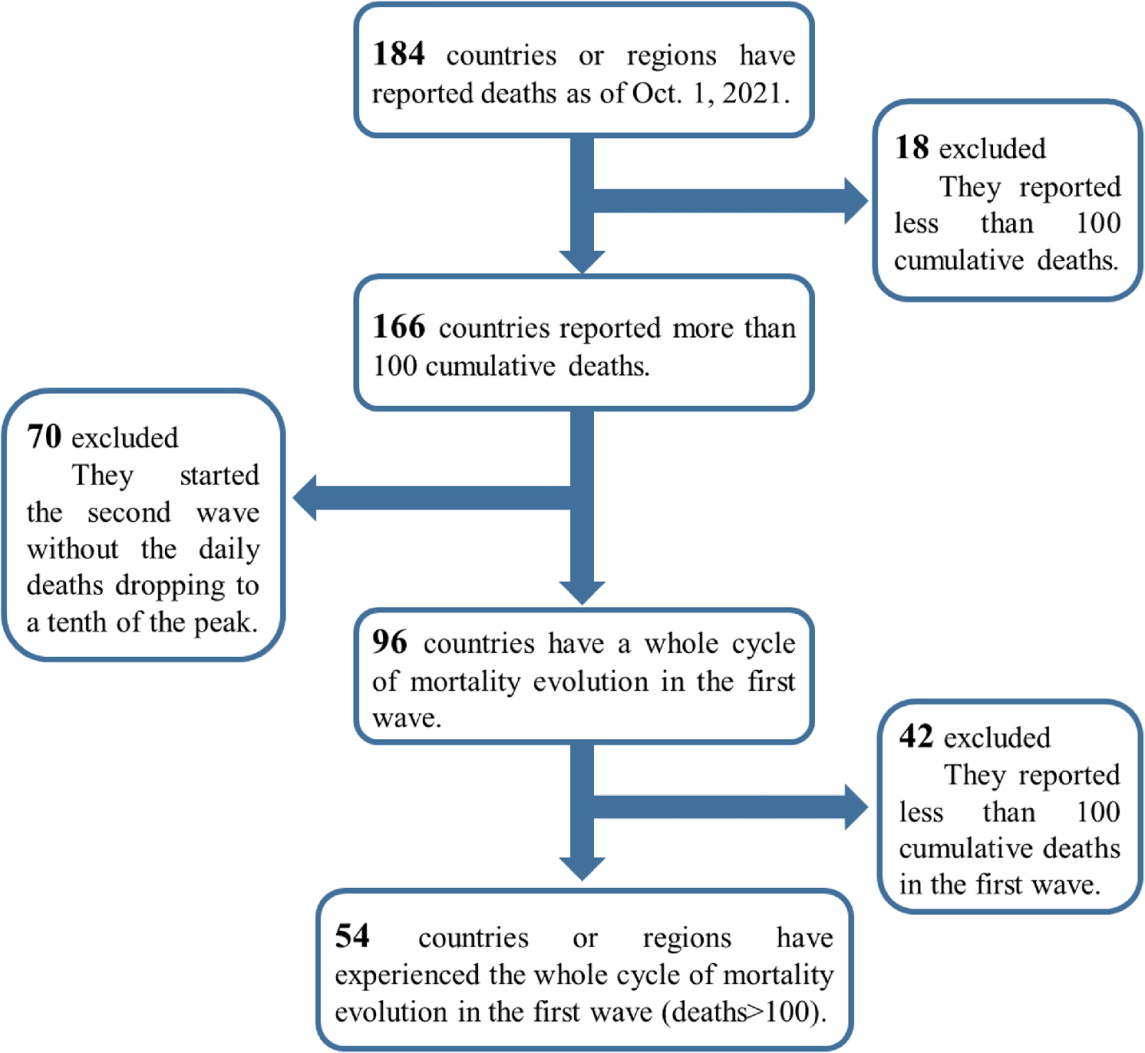
The inclusion criteria of first wave data in this paper.

As of October 1, 2021, 184 countries or regions had reported COVID-19 deaths. Due to technical limitations, government interventions, and multiple corrections of the data, the mortality evolution in some countries shows complex behaviours. For example, in 70 countries, the second wave breaks out before the first wave of the epidemic ends. Since the goal of this study is to analyse the complete evolution of mortality in the first wave, we propose the explicit inclusion criterion that only countries with post-peak daily deaths falling to less than one-tenth of the peak should be considered a qualified sample. Although the model provides a good description of mortality for some countries with total deaths less than 100 (see Supplementary Information), we conservatively quantify samples greater than 100, for statistical significance and clarity of the criteria. Finally, 54 countries or regions were included in this study (see Fig. 5). In addition, we have selected 17, 11, and 10 countries, within the 54 countries, that have a clear and complete evolution in the second, third, and fourth waves respectively.

### Data and metrics

The data used in our study all come from open-access databases. Specifically, the COVID-19 case data are aggregated from the Johns Hopkins University database^26^ (as of June 10, 2022), while the data of Wuhan are from the Health Commission of Hubei Province^27^. The population, median age, and hospital beds (per 1000 population) are from the database of Our World in Data^28^. For China, we chose the data of Wuhan City (Hubei Province, China) instead of national data because of Wuhan’s comparable population size and pandemic scale. The population and bed data in Wuhan are from the Wuhan Statistics Bureau^29^. It is worth mentioning that the data of these state-level variables are updated to the most recent year but often not to the same year.

### Bed occupancy

We propose the “Bed occupancy” to evaluate the availability of hospital beds across countries, which is calculated by dividing the number of hospitalized cases by the number of beds at the peak of the epidemic. Here, “the number of hospitalized cases” is calculated by the following relation: hospitalized cases = confirmed − recovered − deaths.

### The epidemic duration

The epidemic duration is defined to eliminate the long-tail effect when comparing different epidemic waves across countries. Using the peak of daily new deaths as a reference, we define the date when the new deaths climb to more than one-tenth of the peak as the outbreak date (${t}_{outbreak}$) and the date when the new deaths fall below one-tenth of the peak as the saturation date (${t}_{saturation}$). The period between the outbreak date and the saturation date is then defined to be the duration of the epidemic. A 10-day smoothing is carried out on the daily new deaths to obtain a smooth curve.

### Model and parameters

In the early stages of the outbreak, the number of deaths increased almost exponentially due to weak intervention. With the expansion of the death toll, the social system takes actions to slow down the growth of mortality, such as increasing available medical resources, controlling the spread of the epidemic, and protecting high-risk groups. Eventually, the death toll tends to be saturated in the late stages of the epidemic. Therefore, we use the logistic model to fit the COVID-19 mortality (deaths per 100,000 population, $S(t)$):

1 $$\begin{array}{c}\frac{dS\left(t\right)}{dt}=\frac{1}{\tau }S\left(t\right)\left(1-\frac{S\left(t\right)}{{s}_{0}}\right)\end{array}$$

The solution to Eq. (1) can be written as:

2 $$\begin{array}{c}S\left(t\right)=\frac{{s}_{0}}{1+{e}^{\frac{\left({t}_{c}-t\right)}{\tau }}}\end{array}$$

${s}_{0}$—When $t\gg 0$, $S\left(t\right)\approx {s}_{0}$. Therefore, ${s}_{0}$ denotes the saturation mortality in the late stage of the epidemic.

${t}_{c}$—When $t={t}_{c}$, $S\left(t\right)={s}_{0}/2$. Therefore, ${t}_{c}$ denotes the characteristic time when mortality reaches half of the saturation mortality.

$\tau$—When $t\approx 0$, $S\left(t\right)\ll {s}_{0}$, Eq. (1) can be written as ${\tau }^{-1}\approx {S\left(t\right)}^{^{\prime}}/S\left(t\right)$. Therefore, ${\tau }^{-1}$ denotes the exponential growth rate of mortality, while $\tau$ is the characteristic time for death growth in the early stage of the epidemic.

### The dimensionless coefficient $k$

When $t={t}_{outbreak}$ or $t={t}_{saturation}$, Eq. (2) can be written as:

3.1 $$\begin{array}{c}{t}_{outbreak}={t}_{c}-\mathrm{ln}\left(\frac{{s}_{0}}{S\left({t}_{outbreak}\right)}-1\right)*\tau \end{array}$$

3.2 $$\begin{array}{c}{t}_{saturation}={t}_{c}-\mathrm{ln}\left(\frac{{s}_{0}}{S\left({t}_{saturation}\right)}-1\right)*\tau \end{array}$$

Then, the duration can be obtained by subtracting the two equations above:

4 $$\begin{array}{c}Duration={{t}_{saturation}-t}_{outbreak}=k*\tau \end{array}$$

Here, $$k$$ is:

5 $$k = \ln \left( {\frac{{\frac{{{s_0}}}{{S({t_{outbreak}})}} - 1}}{{\frac{{{s_0}}}{{S({t_{saturation}})}} - 1}}} \right)$$

Thus, we can see that the duration is strictly linearly related to $\tau$ by the factor $k$, which is a property of the logistic variation of the death.

### Parameter determination

We used the Curve Fitting Toolbox of MATLAB to fit the reported data across countries to obtain their parameters respectively, based on the analytic Eq. (2). Further, we used the function *predint* to obtain the uncertainty of the parameters at 95% confidence intervals, which showed to be very small (see Supplementary Information).

## Supplementary Information

Supplementary Information.

## Data Availability

All the data and code used in this study are publicly available at https://github.com/zhanglei-pku/Model-COVID-19-mortality.
