# Supplementary material for: The cross-scale correlations between individuals and nations in COVID-19 mortality
Source: Sci Rep. 2022 Aug 16;12:13895. doi: 10.1038/s41598-022-18179-8 (PMC9380985; doi:10.1038/s41598-022-18179-8)
Supplement: Supplementary file 1 — Supplementary Information. [file 41598_2022_18179_MOESM1_ESM.docx]

Supplementary materials

[1. The epidemic duration 1](#_Toc107239586)

[2. 54 areas in the first wave 1](#_Toc107239587)

[3. 17 areas in the second wave 4](#_Toc107239588)

[4. 11 areas in the third wave 5](#_Toc107239589)

[5. 9 areas in the fourth wave 6](#_Toc107239590)

[6. Countries with deaths under 100 6](#_Toc107239591)

[7. Parameter uncertainty 7](#_Toc107239592)

# The epidemic duration

The epidemic duration is defined to prevent the long-tail effect when comparing and analyzing the evolutionary time of the epidemic across countries. Using the peak of daily new deaths as a reference, we define the date when the new deaths climb to more than one-tenth of the peak as the outbreak date ($t_{outbreak}$) and the date when the new deaths fall below one-tenth of the peak as the saturation date ($t_{saturation}$) The time between the outbreak date and the saturation date is the duration of the epidemic. A 10-day smoothing is carried out on the daily new deaths due to the large fluctuations.

**Fig. 1 The epidemic duration of the first wave (ex. Switzerland). a** The daily new deaths and the characteristic date. The black circles are the reported daily new deaths. The blue line is the 10-day smoothing of the daily deaths. The square is the peak of daily new deaths. The regular triangle is the outbreak date when the new deaths climb to more than one-tenth of the peak. The inverted triangle is the saturation date when the new deaths fall below one-tenth of the peak. **b** The cumulative deaths and the duration of the first wave. The black circles are the reported cumulative deaths. The red circles are the data for the duration of the first wave.

# 54 areas in the first wave

54 countries or regions had a full evolutionary cycle (above 100) in the first wave of the epidemic, based on the inclusion criteria we proposed, as shown in **Fig2, 3**.

**Fig. 2 Model fitting in the first wave.** The red point is the officially reported data, and the black line is the fitting result with 95% confidence intervals (blue bands).

**Fig. 3 Model fitting in the first wave.** The red point is the officially reported data, and the black line is the fitting result with 95% confidence intervals (blue bands).

# 17 areas in the second wave

17 countries or regions had a full evolutionary cycle (above 100) in the second wave of the epidemic across above 54 areas, as shown in **Fig 4**.

**Fig. 4 Model fitting in the second wave.** The red point is the officially reported data, and the black line is the fitting result with 95% confidence intervals (blue bands).

# 11 areas in the third wave

11 countries or regions had a full evolutionary cycle (above 100) in the third wave of the epidemic across above 17 areas, as shown in **Fig 5**.

**Fig. 5 Model fitting in the third wave.** The red point is the officially reported data, and the black line is the fitting result with 95% confidence intervals (blue bands).

# 9 areas in the fourth wave

9 countries or regions had a full evolutionary cycle (above 100) in the fourth wave of the epidemic across above 17 areas, as shown in **Fig 6**.

**Fig. 6 Model fitting in the fourth wave.** The red point is the officially reported data, and the black line is the fitting result with 95% confidence intervals (blue bands).

# Countries with deaths under 100

Countries with total deaths of less than 100 are very affected by statistics due to their low number of deaths (see Fig 7), although the model still provides a good description of mortality for them. Therefore, given the statistical significance and the clarity of the criteria, we still quantify that the sample must be greater than 100.

**Fig. 7 Model fitting of countries with deaths under100 (ex. New Zealand and Iceland).** The red point is the officially reported data, and the black line is the fitting result with 95% confidence intervals (blue bands).

# Parameter uncertainty

We used the Curve Fitting Toolbox of MATLAB to fit the reported data across countries to obtain their parameters respectively. Further, we used the function *predict* to obtain the uncertainty of the parameters at 95% confidence intervals, which showed to be very small (see Table 1).

**Table 1 Parameter uncertainty used in this paper**

| Area | wave | $\tau$ | low bound | up bound | $s_{0}$ | low bound | up bound | $t_{c}$ | low bound | up bound |
| --- | --- | --- | --- | --- | --- | --- | --- | --- | --- | --- |
| Afghanistan | 1 | 17.19 | 16.76 | 17.61 | 3.65 | 3.61 | 3.70 | 68.57 | 67.93 | 69.20 |
| Austria | 1 | 8.15 | 7.75 | 8.55 | 7.80 | 7.67 | 7.92 | 26.28 | 25.79 | 26.78 |
| Belgium | 1 | 7.22 | 6.83 | 7.61 | 77.04 | 75.84 | 78.24 | 25.98 | 25.51 | 26.45 |
| Bosnia and Herzegovina | 1 | 8.09 | 7.75 | 8.44 | 3.87 | 3.81 | 3.94 | 30.14 | 29.68 | 30.61 |
| Cameroon | 1 | 19.44 | 18.25 | 20.62 | 1.52 | 1.48 | 1.56 | 60.41 | 58.76 | 62.06 |
| Canada | 1 | 11.59 | 11.05 | 12.13 | 23.28 | 22.91 | 23.66 | 36.58 | 35.90 | 37.25 |
| Cote d'Ivoire | 1 | 20.00 | 19.46 | 20.54 | 0.40 | 0.40 | 0.41 | 65.43 | 64.70 | 66.16 |
| Croatia | 1 | 9.09 | 8.71 | 9.47 | 2.51 | 2.47 | 2.54 | 32.64 | 32.16 | 33.11 |
| Czechia | 1 | 9.16 | 8.34 | 9.97 | 2.86 | 2.78 | 2.93 | 25.82 | 24.84 | 26.79 |
| Denmark | 1 | 9.84 | 9.23 | 10.45 | 9.74 | 9.54 | 9.95 | 30.12 | 29.36 | 30.88 |
| Eswatini | 1 | 14.03 | 13.61 | 14.46 | 9.17 | 9.07 | 9.26 | 53.42 | 52.87 | 53.96 |
| Finland | 1 | 7.84 | 7.48 | 8.20 | 5.38 | 5.30 | 5.46 | 27.00 | 26.55 | 27.44 |
| France | 1 | 7.38 | 6.96 | 7.81 | 41.04 | 40.39 | 41.70 | 25.55 | 25.04 | 26.05 |
| Germany | 1 | 9.49 | 9.08 | 9.90 | 10.08 | 9.95 | 10.21 | 30.83 | 30.33 | 31.33 |
| Greece | 1 | 10.91 | 10.06 | 11.76 | 1.64 | 1.61 | 1.68 | 29.87 | 28.88 | 30.85 |
| Haiti | 1 | 17.43 | 16.48 | 18.38 | 1.60 | 1.56 | 1.64 | 60.03 | 58.65 | 61.42 |
| Hungary | 1 | 11.27 | 10.61 | 11.93 | 5.66 | 5.56 | 5.76 | 32.30 | 31.52 | 33.09 |
| India | 1 | 34.48 | 34.01 | 34.95 | 11.01 | 10.96 | 11.06 | 122.55 | 121.95 | 123.15 |
| Ireland | 1 | 7.55 | 7.45 | 7.66 | 32.47 | 32.33 | 32.60 | 29.24 | 29.12 | 29.37 |
| Israel | 1 | 7.69 | 7.17 | 8.22 | 3.11 | 3.04 | 3.17 | 24.95 | 24.32 | 25.59 |
| Italy | 1 | 11.58 | 10.95 | 12.21 | 54.30 | 53.41 | 55.19 | 34.65 | 33.89 | 35.40 |
| Japan | 1 | 10.02 | 9.80 | 10.23 | 0.72 | 0.71 | 0.72 | 41.52 | 41.23 | 41.81 |
| Kazakhstan | 1 | 7.25 | 6.37 | 8.13 | 7.95 | 7.58 | 8.32 | 22.84 | 21.69 | 24.00 |
| Korea, South | 1 | 11.13 | 10.81 | 11.45 | 0.51 | 0.50 | 0.51 | 35.70 | 35.29 | 36.10 |
| Luxembourg | 1 | 9.04 | 8.40 | 9.68 | 16.78 | 16.41 | 17.15 | 27.89 | 27.12 | 28.66 |
| Madagascar | 1 | 11.92 | 11.37 | 12.47 | 0.75 | 0.74 | 0.76 | 41.95 | 41.29 | 42.62 |
| Malawi | 1 | 8.43 | 8.07 | 8.79 | 0.85 | 0.83 | 0.86 | 27.59 | 27.14 | 28.05 |
| Malaysia | 1 | 9.19 | 8.30 | 10.08 | 0.34 | 0.33 | 0.35 | 18.45 | 17.51 | 19.39 |
| Mali | 1 | 15.92 | 15.54 | 16.31 | 0.63 | 0.62 | 0.63 | 54.22 | 53.65 | 54.79 |
| Mauritania | 1 | 8.06 | 7.44 | 8.69 | 3.14 | 3.07 | 3.21 | 25.27 | 24.52 | 26.02 |
| Morocco | 1 | 6.58 | 5.96 | 7.19 | 0.48 | 0.47 | 0.49 | 20.16 | 19.44 | 20.89 |
| Namibia | 1 | 10.20 | 9.76 | 10.64 | 4.73 | 4.67 | 4.80 | 36.44 | 35.89 | 36.98 |
| Nepal | 1 | 28.26 | 27.74 | 28.78 | 7.08 | 7.02 | 7.14 | 96.77 | 96.00 | 97.55 |
| Netherlands | 1 | 10.13 | 9.59 | 10.66 | 34.39 | 33.84 | 34.93 | 30.03 | 29.40 | 30.67 |
| Nicaragua | 1 | 24.63 | 24.25 | 25.00 | 2.20 | 2.19 | 2.21 | 55.38 | 54.89 | 55.86 |
| Nigeria | 1 | 24.29 | 24.16 | 24.42 | 0.53 | 0.53 | 0.53 | 65.83 | 65.67 | 65.99 |
| Norway | 1 | 6.22 | 5.84 | 6.60 | 3.88 | 3.82 | 3.93 | 18.85 | 18.41 | 19.29 |
| Pakistan | 1 | 15.30 | 15.02 | 15.57 | 2.80 | 2.78 | 2.82 | 64.59 | 64.22 | 64.96 |
| Portugal | 1 | 12.77 | 11.89 | 13.66 | 14.35 | 14.03 | 14.66 | 34.23 | 33.17 | 35.28 |
| Qatar | 1 | 14.41 | 13.62 | 15.20 | 6.00 | 5.88 | 6.13 | 40.72 | 39.70 | 41.73 |
| Senegal | 1 | 22.71 | 22.27 | 23.16 | 1.86 | 1.85 | 1.88 | 79.35 | 78.74 | 79.97 |
| Serbia | 1 | 10.53 | 10.32 | 10.74 | 3.64 | 3.61 | 3.66 | 28.92 | 28.65 | 29.20 |
| Slovenia | 1 | 7.10 | 6.65 | 7.55 | 4.89 | 4.79 | 5.00 | 25.56 | 24.99 | 26.13 |
| Spain | 1 | 8.94 | 8.30 | 9.57 | 57.77 | 56.56 | 58.97 | 26.82 | 26.07 | 27.58 |
| Sudan | 1 | 12.93 | 12.16 | 13.71 | 1.66 | 1.64 | 1.69 | 49.15 | 48.22 | 50.07 |
| Sweden | 1 | 17.20 | 16.36 | 18.04 | 54.32 | 53.42 | 55.22 | 48.63 | 47.60 | 49.67 |
| Switzerland | 1 | 7.53 | 7.18 | 7.89 | 20.16 | 19.84 | 20.48 | 26.68 | 26.23 | 27.13 |
| United Arab Emirates | 1 | 12.15 | 10.99 | 13.31 | 2.82 | 2.76 | 2.89 | 23.09 | 21.91 | 24.27 |
| United Kingdom | 1 | 10.72 | 10.09 | 11.36 | 55.20 | 54.24 | 56.16 | 31.36 | 30.60 | 32.12 |
| Uzbekistan | 1 | 23.85 | 23.06 | 24.64 | 1.77 | 1.75 | 1.79 | 68.73 | 67.70 | 69.77 |
| Yemen | 1 | 14.66 | 13.95 | 15.38 | 1.81 | 1.78 | 1.84 | 46.03 | 45.15 | 46.91 |
| Zambia | 1 | 10.27 | 8.13 | 12.41 | 1.48 | 1.31 | 1.66 | 22.46 | 19.16 | 25.76 |
| Zimbabwe | 1 | 10.84 | 10.15 | 11.53 | 1.56 | 1.51 | 1.61 | 40.82 | 39.73 | 41.91 |
| Wuhan | 1 | 6.19 | 6.01 | 6.37 | 21.88 | 21.69 | 22.07 | 23.93 | 23.71 | 24.15 |
| Afghanistan | 2 | 15.45 | 15.19 | 15.71 | 2.45 | 2.43 | 2.47 | 63.69 | 63.32 | 64.07 |
| Eswatini | 2 | 8.64 | 8.29 | 8.99 | 44.70 | 44.06 | 45.33 | 31.74 | 31.29 | 32.19 |
| Madagascar | 2 | 19.06 | 18.90 | 19.21 | 2.30 | 2.29 | 2.30 | 51.54 | 51.34 | 51.73 |
| Malawi | 2 | 6.94 | 6.61 | 7.27 | 4.42 | 4.35 | 4.49 | 25.93 | 25.52 | 26.35 |
| Mali | 2 | 11.50 | 11.09 | 11.91 | 0.98 | 0.97 | 0.99 | 40.46 | 39.94 | 40.99 |
| Mauritania | 2 | 8.18 | 7.79 | 8.57 | 5.27 | 5.18 | 5.35 | 29.82 | 29.33 | 30.31 |
| Morocco | 2 | 31.64 | 31.21 | 32.07 | 23.32 | 23.19 | 23.45 | 109.22 | 108.64 | 109.80 |
| Senegal | 2 | 20.69 | 20.24 | 21.13 | 4.72 | 4.68 | 4.76 | 71.04 | 70.44 | 71.63 |
| Serbia | 2 | 11.57 | 11.36 | 11.77 | 6.93 | 6.89 | 6.97 | 30.79 | 30.54 | 31.03 |
| Slovenia | 2 | 20.76 | 19.96 | 21.55 | 179.78 | 177.53 | 182.02 | 59.87 | 58.90 | 60.83 |
| Sweden | 2 | 18.19 | 17.55 | 18.84 | 72.52 | 71.41 | 73.62 | 59.10 | 58.20 | 60.00 |
| Switzerland | 2 | 20.93 | 20.75 | 21.11 | 95.63 | 95.33 | 95.93 | 57.43 | 57.20 | 57.66 |
| Yemen | 2 | 11.57 | 11.26 | 11.88 | 2.32 | 2.30 | 2.34 | 43.57 | 43.19 | 43.95 |
| Zambia | 2 | 13.12 | 12.59 | 13.65 | 4.47 | 4.41 | 4.52 | 36.62 | 35.99 | 37.25 |
| Zimbabwe | 2 | 6.35 | 6.08 | 6.61 | 7.25 | 7.16 | 7.33 | 24.08 | 23.76 | 24.39 |
| Denmark | 2 | 13.39 | 13.25 | 13.53 | 28.51 | 28.39 | 28.64 | 55.17 | 54.97 | 55.37 |
| India | 2 | 16.03 | 15.55 | 16.51 | 19.28 | 19.14 | 19.41 | 55.02 | 54.46 | 55.57 |
| Afghanistan | 3 | 12.44 | 12.01 | 12.87 | 11.16 | 11.01 | 11.31 | 44.23 | 43.65 | 44.81 |
| Eswatini | 3 | 11.93 | 11.67 | 12.18 | 47.73 | 47.37 | 48.10 | 43.89 | 43.56 | 44.22 |
| Madagascar | 3 | 11.25 | 10.58 | 11.92 | 1.43 | 1.40 | 1.46 | 39.84 | 38.95 | 40.72 |
| Malawi | 3 | 10.27 | 10.03 | 10.51 | 5.71 | 5.67 | 5.75 | 42.90 | 42.61 | 43.20 |
| Mali | 3 | 10.64 | 10.25 | 11.04 | 0.82 | 0.80 | 0.83 | 41.15 | 40.55 | 41.76 |
| Mauritania | 3 | 12.57 | 12.00 | 13.15 | 6.28 | 6.15 | 6.42 | 41.61 | 40.76 | 42.46 |
| Morocco | 3 | 11.68 | 11.38 | 11.99 | 13.94 | 13.83 | 14.05 | 44.32 | 43.94 | 44.69 |
| Senegal | 3 | 10.13 | 9.86 | 10.39 | 4.14 | 4.10 | 4.18 | 40.71 | 40.35 | 41.06 |
| Switzerland | 3 | 14.36 | 13.61 | 15.10 | 4.80 | 4.66 | 4.93 | 45.14 | 43.96 | 46.32 |
| Yemen | 3 | 17.16 | 16.40 | 17.92 | 1.94 | 1.91 | 1.97 | 57.10 | 56.11 | 58.10 |
| Zambia | 3 | 9.18 | 8.80 | 9.56 | 12.13 | 11.99 | 12.27 | 29.73 | 29.28 | 30.18 |
| Zimbabwe | 3 | 9.38 | 9.07 | 9.69 | 18.82 | 18.64 | 18.99 | 38.24 | 37.86 | 38.62 |
| Afghanistan | 4 | 9.59 | 9.29 | 9.90 | 0.76 | 0.75 | 0.77 | 42.01 | 41.59 | 42.44 |
| Eswatini | 4 | 6.86 | 6.57 | 7.16 | 11.04 | 10.87 | 11.20 | 26.95 | 26.57 | 27.33 |
| Malawi | 4 | 9.45 | 9.03 | 9.86 | 1.58 | 1.56 | 1.60 | 30.93 | 30.43 | 31.43 |
| Mali | 4 | 22.18 | 20.83 | 23.52 | 0.89 | 0.85 | 0.93 | 62.51 | 60.01 | 65.01 |
| Mauritania | 4 | 10.65 | 10.30 | 11.00 | 2.96 | 2.92 | 3.00 | 36.60 | 36.11 | 37.08 |
| Morocco | 4 | 8.67 | 8.53 | 8.81 | 3.28 | 3.26 | 3.30 | 36.07 | 35.88 | 36.25 |
| Switzerland | 4 | 35.73 | 34.96 | 36.50 | 33.76 | 33.42 | 34.11 | 131.57 | 130.43 | 132.72 |
| Zambia | 4 | 7.58 | 7.28 | 7.87 | 1.42 | 1.41 | 1.44 | 26.70 | 26.34 | 27.07 |
| Zimbabwe | 4 | 7.77 | 7.47 | 8.08 | 4.35 | 4.29 | 4.40 | 30.18 | 29.80 | 30.55 |
